# Multi-Frame Content-Aware Mapping Network for Standard-Dynamic-Range to High-Dynamic-Range Television Artifact Removal

**DOI:** 10.3390/s24010299

**Published:** 2024-01-04

**Authors:** Zheng Wang, Gang He

**Affiliations:** School of Telecommunications Engineering, Xidian University, Xi’an 710071, China; jackwu0630@gmail.com

**Keywords:** standard dynamic range (SDR), high dynamic range (HDR), video coding, artifact removal

## Abstract

Recently, advancements in image sensor technology have paved the way for the proliferation of high-dynamic-range television (HDRTV). Consequently, there has been a surge in demand for the conversion of standard-dynamic-range television (SDRTV) to HDRTV, especially due to the dearth of native HDRTV content. However, since SDRTV often comes with video encoding artifacts, SDRTV to HDRTV conversion often amplifies these encoding artifacts, thereby reducing the visual quality of the output video. To solve this problem, this paper proposes a multi-frame content-aware mapping network (MCMN), aiming to improve the performance of conversion from low-quality SDRTV to high-quality HDRTV. Specifically, we utilize the temporal spatial characteristics of videos to design a content-aware temporal spatial alignment module for the initial alignment of video features. In the feature prior extraction stage, we innovatively propose a hybrid prior extraction module, including cross-temporal priors, local spatial priors, and global spatial prior extraction. Finally, we design a temporal spatial transformation module to generate an improved tone mapping result. From time to space, from local to global, our method makes full use of multi-frame information to perform inverse tone mapping of single-frame images, while it is also able to better repair coding artifacts.

## 1. Introduction

Over the past few decades, television production technology has seen rapid advancements. From standard definition (SD) to high definition (HD) and then to ultra-high definition (UHD or 4K, and even 8K), the progress in television and video technology is evident. Central to these advancements is the role of improved sensor technology, which has enabled the capture of richer details and more accurate color reproduction. Similarly, advancements in color gamut and dynamic range have transitioned from the BT.709 standard to the BT.2020 standard, largely due to the capabilities of these advanced sensors. Concurrently, standard dynamic range (SDR) technology has gradually evolved into high dynamic range (HDR) technology, offering audiences a more realistic, dynamic, and color-rich visual experience.

The incorporation of enhanced sensors in modern cameras has been pivotal in HDR technology. They have been implemented in many modern display devices and televisions, providing users with a higher contrast and a richer color representation. However, despite the advancements in sensor technology, the availability of HDR video content remains relatively low. Most of the existing video resources are still provided in SDR format, which to some extent limits consumers’ opportunities to experience HDR display technology. This phenomenon might be attributed to various factors, including but not limited to the cost of producing HDR content and technical requirements. Therefore, while the proliferation of HDR display technology offers the potential for elevating video content quality, the production and distribution of HDR video content still face some challenges.

Upon capturing light, a camera equipped with state-of-the-art sensors performs several processes before displaying the video image on a monitor. Initially, the light signal is transformed into a digital signal via a CMOS sensor. Following this, tone mapping [1] is employed to convert the high-dynamic digital signal captured by the sensor into a low-dynamic signal. Subsequently, gamut mapping adjusts the image color to match the target color gamut. The linear signal is then altered to a nonlinear signal through the optical electronic transfer function (OETF) [2,3]. This digital signal is then quantized and subjected to arithmetic coding [4], making it ready for encoding and decoding through a codec [5,6]. After decoding, the nonlinear signal is reverted to a linear digital signal via an electronic optical transfer function (EOTF), which is then converted into an optical signal for playback on the monitor. The primary distinction between SDR and HDR lies in the utilization of different EOTFs and OETFs, which are crucial in rendering image brightness and color.

HDRTVs, compared to SDRTVs, offer notable advancements in visual perception, among other aspects. However, a significant portion of current video resources remain in SDR format, a historical issue rooted in the capabilities of earlier video recording hardware and sensors that stored videos in the SDRTV format. Given this, devising a solution for converting SDRTV to HDRTV, especially leveraging the data from advanced sensors, is valuable. In this discourse, the process of converting SDRTV to HDRTV is represented as SDRTV-to-HDRTV, aligning with the method mentioned in [7]. SDR-to-HDRTV denotes the conversion from an SDR television image to an HDR television image, where HDR television images, especially those captured with modern sensors, can be displayed on devices through tone mapping.

Earlier approaches [8,9,10] combined super-resolution techniques with SDRTV-to-HDRTV conversion, endeavoring to establish a pipeline to transition from low-resolution SDR video to high-resolution HDR video. In a different vein, HDRTVNET [7] introduced a multi-stage mechanism to achieve SDRTV-to-HDRTV conversion, employing global tone mapping, local image enhancement, and image generation. Similarly, the HDCFM framework [11] involves hierarchical dynamic context feature mapping to facilitate the learning of the mapping function from the SDR frame to the HDR frame.

As mentioned above, historical technical constraints and copyright issues have resulted in a vast quantity of current SDRTV videos lacking nearly lossless versions, leaving only relatively low-quality SDRTV versions available. The practical application of the SDRTV-to-HDRTV method necessitates the conversion of low-quality (LQ) SDRTV to high-quality (HQ) HDRTV. Concurrently, prior research [12,13] discovered that the traditional technique of transitioning from LQ SDRTV to HQ HDRTV tends to magnify the coding artifacts.

**Figure 1:**
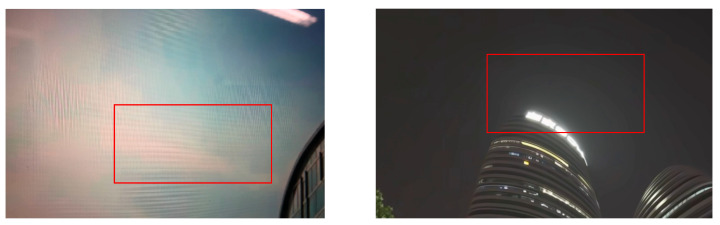
Amplified artifacts when applying the previous SDRTV-to-HDRTV method to low-quality SDR video. The notable artifacts are highlighted within the red rectangles. As shown in the left figure, HDR videos generated by the previous methods will contain amplified blocking artifacts. In the right figure, the banding artifacts are also amplified due to the lack of encoding compression.

In particular, as illustrated in the left figure of Figure 1, applying inverse tone mapping to LQ SDRTV significantly amplifies blocking artifacts. Likewise, the right part reveals how banding artifacts are intensified due to the oversight of encoding compression during the conversion process. These observations confirm that the process of converting SDRTV to HDRTV often exacerbates the encoding artifacts inherent in SDRTV, which in turn diminishes the visual quality of the resultant video. The challenges posed by encoding artifacts are a crucial consideration in developing and refining methods for SDRTV-to-HDRTV conversion in order to attain a superior visual output in the HDRTV format.

In this paper, we present a method to address the challenge of converting low-quality standard-dynamic-range television (LQ-SDRTV) to high-quality high-dynamic-range television (HQ-HDRTV) with a focus on improving the visual quality of the converted video. We propose a multi-frame content-aware mapping network, encompassing temporal-spatial alignment, feature modulation, and quality enhancement to significantly improve the performance of LQ-SDRTV to HQ-HDRTV conversion while simultaneously enhancing visual quality. Through the adoption of dynamic convolutions, hybrid prior extraction, and modulation modules, we demonstrate a robust and structured approach to handle the intricacies involved in SDRTV-to-HDRTV conversion, laying a solid foundation for further research and practical applications in this domain.

The main contributions can be summarized as follows:

- We propose a multi-frame content-aware mapping network (MCMN) which takes into consideration the temporal continuity and spatial features of video frames in a structured manner to improve the performance from low-quality SDRTV to high-quality HDRTV.
- An innovative content-aware temporal spatial alignment module (CTAM) is introduced, employing dynamic deformable convolution to enhance the alignment accuracy of features across different frames and scales. Temporal spatial dynamic convolution (TSDC) adapts its convolution kernels based on the evolving temporal spatial patterns in the video, which is crucial for accurately capturing inter-frame relationships.
- The hybrid prior extraction module (HPEM) is designed to capture the multi-scale information in video content which is crucial for subsequent temporal spatial content-adaptive dynamic modulation.
- The temporal spatial transformation module (TSTM) employs a sequence of temporal spatial dynamic convolutions and mapping modules to perform content-adaptive dynamic modulation. Specifically, a cross-temporal mapping module (CTMM), a local spatial mapping module (LSMM), and a global spatial mapping module (GSMM) are introduced to refine both local and global details within images, leading to improved inverse tone mapping results and enhanced correction of encoding artifacts.

The rest of this paper is organized as follows. In Section 2, we introduce the related works to our proposed MCMN. Section 3 presents the motivation and detailed design of the MCMN for SDRTV-to-HDRTV artifact removal. The implementation and experimental results are demonstrated in Section 4. Finally, we conclude this paper in Section 5.

## 2. Related Work

### 2.1. SDRTV-to-HDRTV

Low dynamic range to high dynamic range (LDR-to-HDR) conversion methods aim to predict the physical brightness of a scene, allowing images to represent a broader spectrum of luminance. This is crucial for capturing scenes with significant light variation. Traditional techniques focus on estimating the light source density, which then aids in further expanding the dynamic range. Earlier methods [14,15,16,17] centered on estimating the light source density, using this as a foundation to broaden the dynamic range. Recent advancements have seen the application of deep learning, specifically deep convolutional neural networks, for this conversion. A notable method [18] introduced in 2020 directly converts LDR images to HDR without intermediate steps. Refs. [19,20] introduce the techniques that specifically target and recover overexposed areas in images. Another intriguing approach introduced in [21,22,23,24] offers a prediction mechanism. It predicts multi-exposure LDR image pairs using just a single LDR image. After this prediction, HDR images are synthesized based on the generated multi-exposure image pairs.

The SDRTV-to-HDRTV conversion approach has only emerged in the last two years. Ref. [8] proposes a GAN-based architecture that jointly achieves super-resolution and SDTV to HDRTV conversion. Ref. [9] proposes a hierarchical GAN architecture to accomplish super-resolution and SDRTV to HDRTV conversion. Ref. [7] proposed a method using global feature modulation, local enhancement, and over-exposure compensation, which achieved the best performance. Ref. [10] proposed a global priors guided modulation network to extract color conformity priors and structural similarity priors that are beneficial for SDRTV-to-HDRTV and SR tasks, respectively. Similarly, the HDCFM framework [11] involves hierarchical dynamic context feature mapping to facilitate the learning of the mapping function from SDR frames to HDR frames. This is achieved through a hierarchical feature modulation module coupled with a dynamic context feature transformation module, providing a structured approach to understanding and transforming visual data from SDR to HDR and enhancing the visual output and potentially paving the way for better utilization of existing SDR video resources in the newer HDRTV format.

### 2.2. Artifact Removal

In terms of artifact removal task, numerous studies [25,26,27,28,29,30,31,32,33,34,35] are dedicated to improving the visual quality of compressed images and videos. ARCNN [25] was pioneering in its use of a CNN to alleviate image compression artifacts. Following this, Zhang et al. [26] introduced the DnCNN, which focused on various aspects of image restoration like denoising, deblocking, and super-resolution.

On the video quality enhancement front, Dai et al. [27] made the first stride by adapting a CNN for post-processing in HEVC intra coding. Building on this, Zhang et al. [28] aimed to replace HEVC’s in-loop filter. On the other hand, Yang et al. [29] aimed to minimize distortion in HEVC by enhancing the quality of I and P/B frames without any encoder modifications. In a novel approach, He et al. [30] introduced a partition-masked CNN that leveraged coding unit size data to optimize network performance. Moreover, Ding et al. [31] proposed a squeeze-and-excitation filtering CNN which was designed as an optional in-loop filter to boost HEVC’s efficiency. Xue et al. [32] devised a task-oriented flow network and employed motion compensation through a flow estimation module to enhance video quality. MFQE [33,34] recognized the significant fluctuations between compressed video frames and introduced a peak quality frame detector to improve videos’ visual appearance. Lastly, to overcome the computational inefficiencies of optical flow, the spatio-temporal deformable fusion method [35] was presented as a solution for enhancing compressed videos.

## 3. Methodology

### 3.1. Overall

Our method can be primarily divided into three components: temporal-spatial alignment, tone mapping modulation, and quality enhancement. This approach aims to significantly improve the performance of converting low-quality SDRTV to high-quality HDRTV while simultaneously enhancing visual quality.

**Figure 2:**
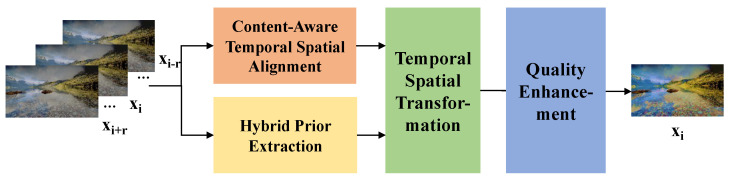
Architecture of our multi-frame content-aware mapping network (MCMN). Specifically, a series of low-quality SDR (LQ-SDR) frames are input to a content-aware temporal spatial alignment module to generate content-aware alignment features. Before proceeding with feature modulation, LQ-SDR frames are sent to the hybrid prior extraction module to yield triplet hybrid priors simultaneously. Next, with the help of a temporal spatial transformation module, the alignment features are tone-mapped into high-dynamic-range features with these hybrid priors. Finally, we employ a quality enhancement module to generate high-quality HDR results.

Given 2r+1 consecutive low-quality SDR frames $X_{\left[i-r:i+r\right]}$, we denote the center frame $X_{i}$ as the target frame that needs to be mapped and the other frames as the reference frames. The input of the network is the target frame $X_{i}$ and the 2r neighboring reference frames, and the output is the enhanced target frame $Y_{i}^{o}$. *X* is a stack of low-quality SDR frames which is defined as

(1) $$X=[X_{i-r},\cdots ,X_{i-1},X_{i},X_{i+1},\cdots ,X_{i+r}],$$

where *i* denotes the frame index and *r* is the maximum range number of reference frames. The architecture of the MCMN is shown in Figure 2. In the following subsection, we will a present a detailed analysis of the motivation behind and rationality of each module.

### 3.2. Content-Aware Temporal Spatial Alignment Module

**Figure 3:**
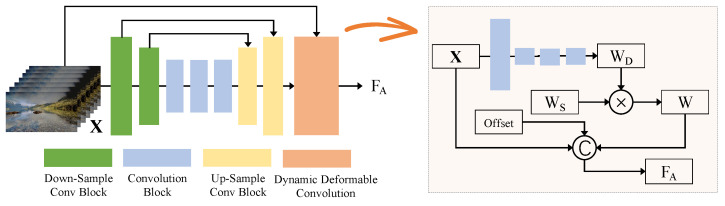
Structure of the content-aware temporal spatial alignment module (CTAM). By introducing learnable dynamic weights, dynamic deformable convolution is proposed to perform content-aware temporal spatial alignment.

When addressing the task of video conversion, we require a profound understanding of the temporal spatial correlation between adjacent video frames. To effectively harness this correlation, we introduce the content-aware temporal spatial alignment module (CTAM). The structure of the CTAM is shown in Figure 3. Considering the efficacy of prior deformable convolution [36] in video frame alignment tasks, we propose a dynamic deformable convolution (DDC) for the initial alignment in this task. We first employ the UNet [37] structure to extract feature offset with temporal spatial information, which can capture subtle changes in image features across different scales, thereby enhancing alignment accuracy. Specifically, the UNet-based offset extraction part consists of two down-sampling convolutional blocks, three convolutional blocks, and three up-sampling convolutional blocks. The number of neurons is 32 in all layers.

To adaptively align these features from different temporal and spatial scales in a content-aware manner, we design a dynamic deformable convolution (DDC). Contrasting this with the standard deformable convolution [36], where only static weights are leveraged, DDC is engineered to determine content-aware characteristic features by introducing dynamic weights. This enables content-based temporal spatial alignment using the initial static weights. In DDC, the regular sampling grid R={(−1,−1),(−1,0),⋯,(0,0),⋯,(0,1),(1,1)} is defined as the 3×3 convolution kernel with dilation 1. Here, the UNet-based offset extraction part is used to gather temporal spatial clues between $X_{i-r}$ and $X_{i+r}$ at diverse scales and generate the sampling parameters △P of the convolution kernel. It can be described as

(2) $$△P=UNet(\left[X_{i-r},\cdots ,X_{i-1},X_{i},X_{i+1},\cdots ,X_{i+r}\right]),$$

where $△P=\{△p_{k}|k=1,\cdots ,|R|\}$ is the collection of learnt predicted offsets $△p_{k}$.

Many variants of DConv focus on finding better ways to enhance the offset. It should be noted that the weights of standard deformable convolution are static. Therefore, we introduce dynamic weights $W_{D}$ which are learned from the original input directly. Here, we utilize cascaded global pooling, a 1×1 convolutional layer, and the sigmoid activation function to obtain the dynamic weights $W_{D}$. The content-adaptive weights are the combination of the static and dynamic weights in a dot multiplication. The aligned features $F_{CTAM}$ from DDC at location $p_{0}$ can be computed:

(3) $$F_{CTAM}\left(p_{0}\right)=\sum_{p_{k}\in R}\left(W_{k}^{S}⊗W_{k}^{D}\right)\cdot X\left(p_{0}+p_{k}+△p_{k}\right),$$

The convolution will be performed on the deformed sampling locations $p_{k}+△p_{k}$, where $W_{k}^{S}$, $W_{k}^{D}$, $p_{k}$, and $△p_{k}$ denote the static weights, dynamic weights, pre-specific offset, and learnt offset for *k*-th location in R. ⊗ denotes dot multiplication.

After this, the content-adaptive temporal-spatial feature alignment features can be acquired. The success of this alignment process is crucial for subsequent tasks, as it aids in precisely capturing the temporal-spatial relationship between video frames, laying a solid foundation for feature modulation and recovery.

### 3.3. Hybrid Prior Extraction Module

In the field of video processing, features extracted from SDR video frames typically reside in the SDR feature space, while those extracted from HDR video frames are in the HDR feature space. As a result, the conversion from SDRTV to HDRTV can be modeled as a feature mapping. Correspondingly, previous works on SDRTV-to-HDRTV primarily focused on processing individual frames, obtaining low-dynamic-range features from SDR frames via a convolutional neural network, then mapping these features to high-dynamic-range features and finally restoring them to the HDR image space. Our approach is based on video, and fully exploits the spatio-temporal characteristics of videos. Hence, we introduce the spatio-temporal transformation module, aimed at capturing the spatio-temporal characteristic of videos, thereby achieving a superior conversion result.

**Figure 4:**
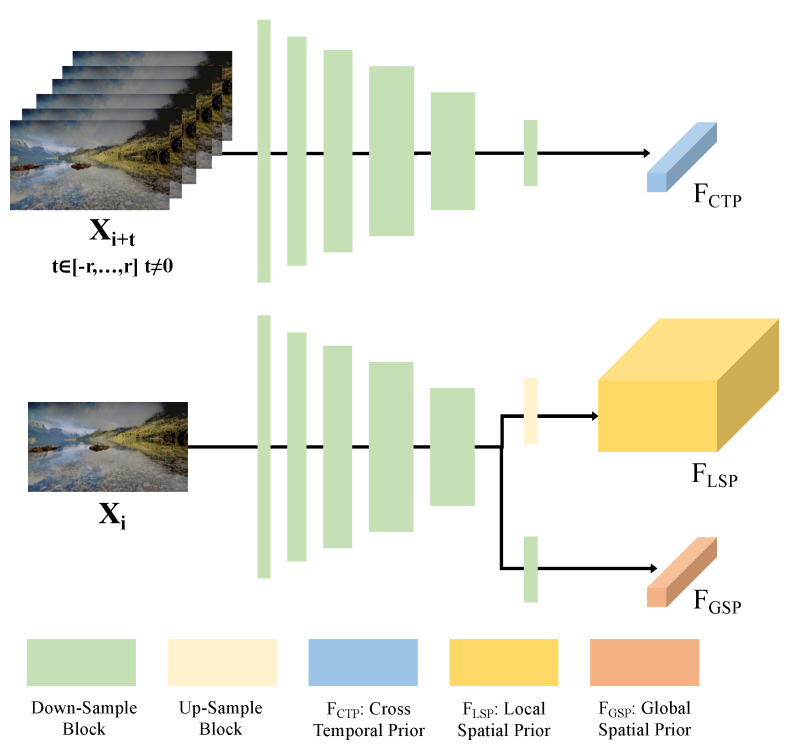
Structure of the hybrid prior extraction module (HPEM). In the cross-temporal prior branch, the adjacent reference frames ($X_{i+t},t\in [$-r,⋯,r],t≠0) are used to generate the cross-temporal prior using HEMP. In the local spatial and global spatial prior extraction branches, the local spatial prior and global spatial prior are generated using a single target center frame $X_{i}$ simultaneously.

Before proceeding with feature mapping, it is essential to extract mapping priors. To accommodate the unique requirements of our task, we design a hybrid prior extraction module (HPEM), specifically incorporating the cross-temporal prior extraction branch, local spatial prior extraction branch, and global spatial prior extraction branch. The detailed structure of the HPEM is shown in Figure 4. In the context of this module, we input the adjacent reference frames ($X_{i+t},t\in [$-r,⋯,r],t≠0) of the current target frame to the prior extraction module to obtain the cross-temporal prior $F_{CTP}$.

(4) $$F_{CTP}=HPEM\left(X_{i+t}\right),\;t\in \left[-r,\cdots ,r\right],\;t\neq 0,$$

where HPEM denotes the hybrid prior extraction module. Specifically, five down-sample blocks are employed to generate down-sampled features $F_{D}$. Each block is composed of 1×1 conv, average pooling with a stride of 2, and LeakyReLU activation. The numbers of neurons in the five down-sampling blocks are 16, 32, 64, 128, and 128, respectively. For the cross-temporal prior $F_{CTP}$, adaptive pooling is used to yield the temporal weights.

The current target frames are simultaneously individually processed by the HPEM to yield both the local spatial prior $F_{LSP}$ and the global spatial prior $F_{GSP}$. The first part of this process involves distilling spatial information, and thus, the HPEM is employed to generate $F_{LSP}$ and $F_{GSP}$ simultaneously.

(5) $$F_{LSP},\;\;F_{GSP}=HPEM\left(X_{i}\right).$$

Specifically, for the local spatial prior ($F_{LSP}$), which targets pixel-level mapping, we employ a bilinear up-sampling operator to upscale the down-sampled features $F_{D}$ to match the resolution of the input image. Conversely, for the global spatial prior ($F_{GSP}$), which is intended for frame-level mapping, we apply an average pooling operator to further down-sample the down-sampled features $F_{D}$. This process yields one learned global weight for each frame, facilitating effective global spatial mapping. This nuanced handling of spatial priors at different scales is critical for the fidelity of our spatial mapping operations.

The hybrid prior extraction module can adeptly capture multi-scale information of video content, preparing for the subsequent temporal spatial content-adaptive dynamic modulation.

### 3.4. Temporal Spatial Transformation Module

**Figure 5:**
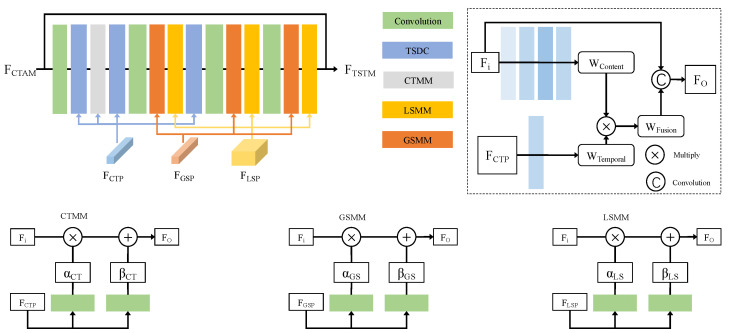
Structure of the temporal spatial transformation module (TSTM). It integrates a sequence of temporal spatial dynamic convolutions (TSDCs), a cross-temporal mapping module (CTMM), a local spatial mapping module (LSMM), and a global spatial mapping module (GSMM) to obtain better tone mapping results.

In the process of converting SDRTV to HDRTV, it is insufficient to process pixels from varied spatial locations identically due to the nuances they present. For example, a frame might contain both overexposed and underexposed areas, requiring tailored processing strategies for each exposure condition. To address this challenge, we develop a temporal spatial transformation module (TSTM) that is adaptive to spatio-temporal content. As shown in Figure 5, this method integrates a sequence of temporal spatial dynamic convolutions (TSDCs), a cross-temporal mapping module (CTMM), a local spatial mapping module (LSMM), and a global spatial mapping module (GSMM). Next, we will introduce each part in detail.

Temporal spatial dynamic convolution (TSDC) can adeptly modify its convolution kernels in response to the evolving spatio-temporal patterns in the video, aiding in capturing the inter-frame relationships more accurately. Specifically, we utilize the cross-temporal prior to generate the temporal weight $W_{Temporal}$ which contains a plethora of useful temporal-spatial information. The derived $W_{Temporal}$ is then fused with the original content weights $W_{Content}$ to obtain the fused weight $W_{Fusion}$. Finally, the input features are convoluted with the temporal spatial dynamically corrected weight to yield the feature output. This process can adaptively adjust the convolution kernel based on the input data to better capture the dynamic information within videos. The TSDC operator can be formulated by the following equation:

(6) $$F_{o}\left(p_{0}\right)=\sum_{p_{k}\in R}\left(W_{content}⊗W_{temporal}\right)\cdot F_{i}\left(p_{0}+p_{k}\right).$$

After TSDC, we propose the cross-temporal mapping module (CTMM) to perform tone mapping in the temporal dimension. Therefore, we revisit the spatial feature transform (SFT) [10,38] and global feature modulation (GFM) [7,39]. Inspired by the SFT and GFM, the proposed CTMM generates a pair of ($\alpha_{CT},\beta_{CT}$) by definition priors and performs modulations through scaling and shifting, respectively. The CTMM can be formulated by:

(7) $$F_{o}\left(F_{i}|F_{CTP}\right)=\alpha_{CT}⊗F_{i}⊕\beta_{CT},$$

where ⊗ refers to element-wise multiplication and ⊕ is element-wise addition. $F_{i}$ and $F_{o}$ are the input and output of the CTMM.

Moreover, spatial feature modulations, both local and global, focus on refining the details within images, leading to an improved inverse tone mapping result and a heightened ability to rectify encoding artifacts. The processes can be formulated by

(8) $$\begin{array}{c} F_{o}\left(F_{i}|F_{GSP}\right)=\alpha_{GS}⊗F_{i}⊕\beta_{GS},\\ F_{o}\left(F_{i}|F_{LSP}\right)=\alpha_{LS}⊗F_{i}⊕\beta_{LS}, \end{array}$$

where the pairs of ($\alpha_{GS},\beta_{GS}$) and ($\alpha_{LS},\beta_{LS}$) by definition map and perform modulations through scaling and shifting in the global spatial mapping module and the local spatial mapping module.

### 3.5. Quality Enhancement Module

**Figure 6:**
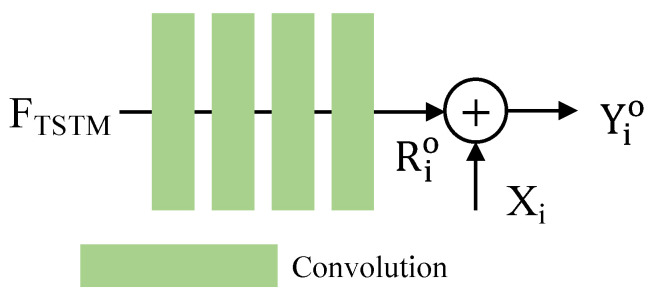
Structure of the quality enhancement module (QEM). We employ a cascade of convolutional layers with residual learning to yield the final enhanced high-quality HDR frame $Y_{i}^{o}$.

Encoding compression will lead to a deterioration in video quality, manifesting as blurring, block artifacts, or other visible compression distortions. To eliminate the artifacts by encoding compression, we introduce a quality enhancement module (QEM) at the final stage. Here, we take advantage of residual learning to generate the results. As illustrated in Figure 6, the module starts by applying a cascade of convolutional layers (here, we employ four layers) to extract the high-frequency information, denoted as the residual $R_{i}^{o}$. The final output, an enhanced high-quality HDR frame $Y_{i}^{o}$, is then obtained by residual learning. The process is formulated as follows:

(9) $$\begin{array}{c} R_{i}^{o}\;=QEM(F_{TSTM}),\\ Y_{i}^{o}\;=X_{i}+R_{i}^{o}. \end{array}$$

The primary idea of the QEM is to fully explore the complementary information within the fused feature maps and accordingly generate an enhanced high-quality HDR frame $Y_{i}^{o}$. Without bells and whistles, this straightforward QEM is capable of achieving satisfying enhancement results.

## 4. Results

In this section, we show the performance of the proposed MCMN. Section 4.1 introduces the training dataset and implementation details. In Section 4.2, we present the quantitative performance of the MCMN. Section 4.3 presents the qualitative performance. Moreover, the ablation study in Section 4.4 is demonstrated to prove the effectiveness of the designed architecture.

### 4.1. Experimental Settings

#### 4.1.1. Dataset

We employ the well-used HDRTVNET dataset [7] as our benchmark. We use X265 [6] to encode SDR videos with different quantization parameters (QPs) (27, 32, 37, 42) to process the videos with different degrees of coding degradation. We compute the multi-scale structural similarity index (MS-SSIM) [40] of adjacent frames for scene segmentation. The MS-SSIM can evaluate the quality of video frames at multiple scales. This feature is crucial for capturing finer details and nuances that are especially relevant in HDR content.

#### 4.1.2. Implementation Details

All experiments were conducted using PyTorch 1.6.0, Python 3.8, CUDA 10.1. The server was equipped with an Intel Core i9-13900K CPU and an NVIDIA GeForce RTX 4090 GPU. In our architecture, the range number *r* for the reference frame is 3. This configuration results in a total of seven input frames being considered for processing. Except for the previous special instructions, the number of neurons in all convolutional layers is 64. Moreover, the ReLU activation function is consistently used across these layers. During the training phase, we employ the SDR video with a quantization parameter (QP) of 37 as input data, and the output is a high-quality HDR video. The Adam optimizer, as mentioned in [41], is employed with an initial learning rate of 0.0005. After reaching 100,000 iterations, the learning rate is adjusted to halve every 60,000 iterations, totaling up to 660,000 iterations for the entire training process. We use L1 Loss between the high-quality high-dynamic standard image $Y_{i}^{HQ-HDR}$ and the enhanced output $Y_{i}^{o}$ of our MCMN to supervise network training. The loss function can be formulated as follows:

(10) $$L={∥Y_{i}^{HQ-HDR}-Y_{i}^{o}∥}_{1}.$$

To verify the performance of different algorithms in a fair generalization, we take the last six stored weights to test the metrics. The model trained on coding degradation with a fixed QP = 37 was tested on four different QP coding test sets. As with [42], this multiple evaluation ensures that we can accurately and fairly evaluate the performance of different models.

### 4.2. Quantitative Results

The table presents a quantitative comparison of various image quality enhancement methods; specifically, it details their performance in terms of the peak signal-to-noise ratio (PSNR) and structural similarity index measure (SSIM) at different quantization parameter (QP) values. The PSNR is a widely used metric to measure the quality of reconstructed images, with higher values indicating a better quality. Similarly, the SSIM is another crucial measure that evaluates the perceptual quality of images by comparing structural similarities. We compare the proposed method with state-of-the-art SDRTV-to-HDRTV methods (FMNet [43], HDRUNET [44], HDCFM [11], HyCondITM [45], etc.). For a fair comparison, each method was retrained on the same training set. The last six checkpoints of each model were then tested on a common test set, and the average PSNR value was derived.

The quantitative results for each metric are shown in Table 1. The mean PSNR of our method is 33.208, while the mean PSNR of previous methods varies from 21.826 dB to 33.100 dB. It can be observed that our method consistently outperforms all comparison methods in terms of the mean PSNR for the test set, highlighting its robustness and superior performance. The difference in performance between the proposed method and the previous state-of-the-art (SOTA) method is notable. Specifically, comparing the proposed method with the preceding best performer, there is an improvement of 0.18 dB in the PSNR metric when the QP is set to 27. The proposed method showcases a superior performance in enhancing image quality across various quantization parameters.

Moreover, the superiority of our method extends to the SSIM as well, where it leads the pack among all the compared methods. This achievement in SSIMs, coupled with the high PSNR values, offers a more comprehensive assessment of our method’s performance. By excelling in both objective metrics like the PSNR and subjective metrics like the SSIM, our method presents itself as a potentially pioneering solution in the realm of image quality enhancement, offering improvements from both objective and subjective perspectives.

### 4.3. Qualitative Results

**Figure 7:**
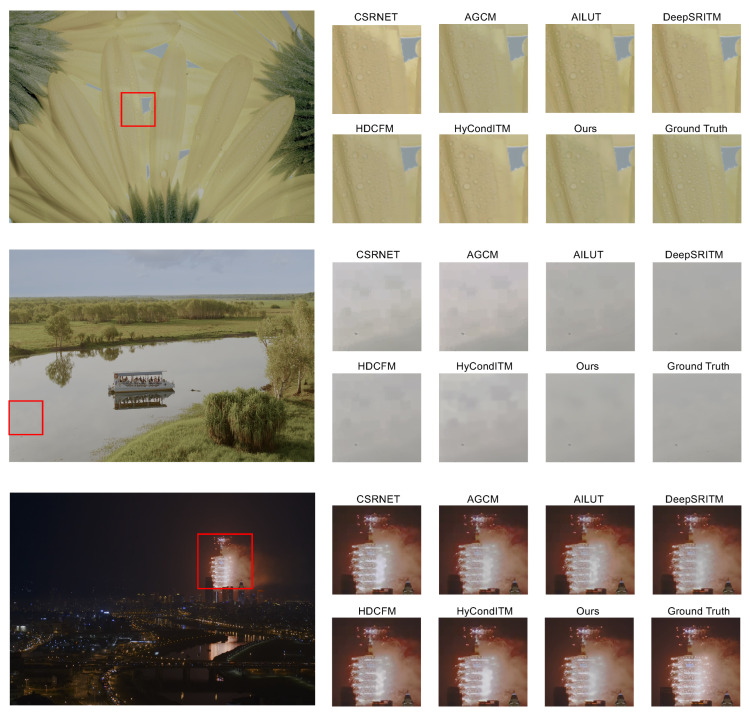
Qualitative results. Our method produces results with fewer artifacts and of a higher quality than previous methods (CSRNET [39], ACGM [7], AILUT [46], DeepSRITM [8], HDCFM [11], and HyCondITM [45]). Ground truth represents the reference standard against which the quality of our method’s output is compared. The areas enclosed by the red rectangles are magnified for better observation.

Figure 7 presents the subjective comparison between our method and previous methods. In the first image, our approach can map the color of sunflower petals to the high-dynamic-range space with greater precision. Observing the second image, we can see that the artifacts in the lake section of the sample image are amplified by methods such as CSRNET [39], ACGM [7], HDCFM [11], and HyCondITM [45]. However, our technique effectively eliminates these artifacts caused by compression, bringing the image closer to the ground truth. In the third image, compared to other methods, our approach is able to restore the original image to a greater extent.

### 4.4. Ablation Study

In this section, we will perform an ablation study to verify the effectiveness of the each part of our multi-frame content-aware mapping network (MCMN) design.

#### 4.4.1. Ablation Study of the Content-Aware Temporal Spatial Alignment Module (CTAM)

To verify the effectiveness of our CTAM module, we conducted ablation studies. In this context, Exp. 1 denotes a baseline without any spatial temporal alignment, Exp. 2 denotes using deformable convolution for temporal alignment operations, and *MCMN* denotes a structure that utilizes dynamic deformable convolution for content-aware temporal spatial alignment. The results are demonstrated in Table 2. Without any temporal spatial alignment, as seen in the Exp. 1, the model’s average PSNR stands at 32.390 dB. When the DCN is introduced for temporal alignment, there is a rise in the PSNR, reaching 32.689 dB. Most significantly, with the incorporation of the CTAM for content-aware temporal-spatial alignment in the model, the PSNR peaks at a value of 33.208 dB. This shows that our method effectively extracts spatial temporal information, aiding the model in tone mapping and artifacts.

#### 4.4.2. Ablation Study of the Temporal Spatial Transformation Module (TSTM)

To validate the efficacy of individual components within the temporal spatial transformation module (TSTM), we undertook a comprehensive ablation study. This analysis spanned the global spatial mapping module (GSMM), the local spatial mapping module (LSMM), the cross-temporal mapping module (CTMM), and temporal spatial dynamic convolutions (TSDC), with a focus on adaptively reconstructing high-dynamic-range features.

Global spatial mapping module (GSMM) performance. When only the global spatial mapping module (GSMM) is employed (without the LSMM, the CTMM, and TSDC), the average PSNR is 32.390 dB. This serves as our baseline metric for performance.

Local spatial mapping module (LSMM) impact. Incorporating the LSMM alongside the GSMM (without the CTMM and TSDC) improves the average PSNR to 32.748 dB. This increment indicates the value of local spatial mapping in enhancing video quality.

Cross-temporal mapping module (CTMM) impact. Engaging the plain CTMM (only with the GSMM) further elevates the PSNR to 32.847 dB. This underscores the significance of temporal tone mapping. Notably, when introducing the LSMM simultaneously, the performance of the mean PSNR soared to 33.120 dB, marking a significant improvement of 0.273 dB. This finding reinforces the complementary nature of local and global and intra-frame and inter-frame information, collaboratively enhancing the method’s performance.

**Figure 8:**
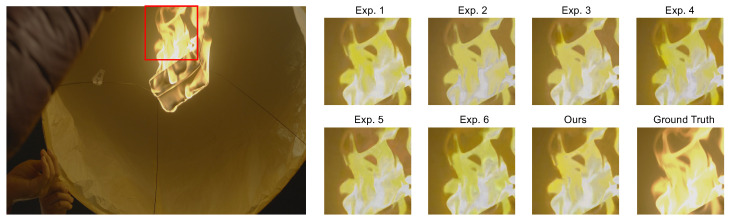
Qualitative results of the ablation study. Compared with the ablation variants (Exp. 1–6), our MCMN effectively eliminates artifacts and achieves color reproduction to closely match the ground truth. Ground truth represents the reference standard against which the quality of our method’s output is compared. The areas enclosed by the red rectangle are magnified for better observation.

Comprehensive module activation. The highest PSNR value of 33.208 dB was achieved when all modules (the GSMM, the LSMM, the CTMM, and TSDC) are utilized. This combined approach delivers the most optimal performance, with an impressive average PSNR increase of 0.818 dB compared to the baseline. Moreover, we conducted a qualitative comparison as part of our ablation study, shown in Figure 8. Compared with the ablation variants, our MCMN effectively eliminates artifacts and achieves color reproduction that closely matches the ground truth. This visual evidence, alongside the quantitative data, provides a more comprehensive evaluation of each part of our proposed method.

In conclusion, the temporal spatial transformation module (TSTM) plays a pivotal role in enhancing video quality during the SDRTV-to-HDRTV conversion process. Each module within the TSTM contributes uniquely to this enhancement, with their collective use delivering the most superior results.

### 4.5. Processing Time

We evaluated the processing time required for converting SDRTV using our method. All measurements were conducted on an NVIDIA GeForce RTX 4090Ti GPU to ensure a consistent testing environment. The processing time was calculated for a resolution of 256 × 256. As detailed in Table 1, our method achieves a processing time of 3.83 milliseconds (ms), corresponding to a real-time processing speed of approximately 261 frames per second (fps). Notably, our MCMN method is significantly more efficient, reducing the conversion time by over 50% compared to the previous SOTA algorithm, HyCondITM. This marked improvement in processing speed, coupled with enhancements in video quality, highlights the practical benefits of our approach.

Furthermore, we analyzed the processing times for various ablation study variants. Table 2 and Table 3 illustrate that while adding each module increases the processing time, the corresponding performance enhancements are substantial. This demonstrates a favorable trade-off between processing time and performance improvements.

## 5. Conclusions

In conclusion, this paper delineates a comprehensive and innovative methodology for addressing the nuanced task of converting low-quality standard-dynamic-range television (LQ-SDRTV) to high-quality high-dynamic-range television (HQ-HDRTV). The focal point of this endeavor is not only to achieve a superior conversion quality but also to significantly enhance the visual quality of the resultant videos, addressing the prevalent issues associated with existing methods. The proposed multi-frame content-aware mapping network (MCMN) epitomizes a holistic approach towards this objective, including temporal-spatial alignment, feature modulation, and quality enhancement within a coherent framework.

The introduction of the content-aware temporal spatial alignment module (CTAM) underscores a crucial step towards accurately aligning features across various scales and frames, facilitated by dynamic deformable convolution. Combining this with temporal-spatial dynamic convolution (TSDC) lays a robust foundation for capturing the intricate inter-frame relationships inherent in video sequences. The hybrid prior extraction module (HPEM) and the temporal spatial transformation module (TSTM) further accentuate the methodical approach employed in this work. These modules diligently capture multi-scale information and perform content-adaptive dynamic modulation, respectively, thereby addressing both local and global details within images. The resultant enhanced inverse tone mapping and the correction of encoding artifacts signify notable advancements in the SDRTV to HDRTV conversion domain.

The proposed methodology not only showcases the flexibility and scalability of the approach but also heralds a promising avenue for future research and practical applications. Each module within the network embodies a targeted strategy to tackle specific challenges associated with SDRTV to HDRTV conversion, making the methodology adaptable and conducive for further refinements.

From time to space, from local to global, our method makes full use of multi-frame information to perform inverse tone mapping of single-frame images, while it is also able to better repair coding artifacts. The results emanating from this work underscore a significant stride towards bridging the gap between SDRTV and HDRTV technologies, making a compelling case for the adoption and further exploration of the proposed methodology in real-world applications.

## Figures and Tables

**Table 1:** Quantitative comparison with previous methods. For each model on each QP, we selected the last six checkpoints for evaluation and calculated the mean PSNRs and SSIMs for comparison. The processing time (ms) was calculated for a resolution of 256 × 256. Best and second best results are **bold** and underlined, respectively.

Methods	Time	PSNR				Mean-	Mean-
		QP = 27	QP = 32	QP = 37	QP = 42	PSNR	SSIM
CSRNET [39]	0.3	33.598	32.472	31.288	29.946	31.826	0.9518
STDF [35]	3.7	33.978	32.810	31.591	30.163	32.135	0.9455
AGCM [7]	0.4	34.260	33.123	31.878	30.395	32.414	0.9526
AILUT [46]	3.3	34.265	33.058	31.789	30.350	32.366	0.9498
DeepSRITM [8]	7.4	34.688	33.332	31.998	30.483	32.625	0.9515
FMNet [43]	1.4	34.462	33.474	32.146	30.584	32.666	0.9523
HDRUNET [44]	2.9	34.586	33.591	32.262	30.706	32.786	0.9514
HDCFM [11]	2.4	34.897	33.784	32.440	30.929	33.012	0.9538
HyCondITM [45]	8.3	34.860	33.862	32.573	**31.103**	33.100	0.9554
Ours	3.8	**35.072**	**34.026**	**32.651**	31.083	**33.208**	**0.9555**

**Table 2:** Ablation study of the content-aware temporal spatial alignment module (CTAM) in terms of the PSNR (dB). Best results are **bold**. ✓ indicates that the feature or component was enabled, while ✗ signifies that it was not enabled.

Exp.	Baseline	DCN	CTAM	Time	PSNR				Mean
					QP = 27	QP = 32	QP = 37	QP = 42	
1	✓	✗	✗	2.88	34.097	33.133	31.894	30.437	32.390
2	✓	✓	✗	2.95	34.595	33.409	32.137	30.617	32.689
MCMN	✓	✓	✓	3.83	**35.072**	**34.026**	**32.651**	**31.083**	**33.208**

**Table 3:** Ablation study of the temporal spatial transformation module (TSTM) in terms of the PSNR (dB). Best results are **bold**. ✓ indicates that the feature or component was enabled, while ✗ signifies that it was not enabled.

Exp.	GSMM	LSMM	CTMM	TSDC	Time	PSNR				Mean
						QP = 27	QP = 32	QP = 37	QP = 42	
3	✓	✗	✗	✗	3.09	34.097	33.133	31.894	30.437	32.390
4	✓	✓	✗	✗	3.49	34.628	33.498	32.195	30.673	32.748
5	✓	✗	✓	✗	3.43	34.865	33.567	32.252	30.704	32.847
6	✓	✓	✓	✗	3.56	34.977	33.923	32.569	31.012	33.120
MCMN	✓	✓	✓	✓	3.83	**35.072**	**34.026**	**32.651**	**31.083**	**33.208**

## Data Availability

Data are contained within the article.

## References

[1] Mantiuk R., Daly S., Kerofsky L. Display adaptive tone mapping. Proceedings of the SIGGRAPH ’08: ACM SIGGRAPH 2008 Papers.

[2] (2014). High Dynamic Range Electro-Optical Transfer Function of Mastering Reference Displays.

[3] Nagata Y., Ichikawa K., Yamashita T., Mitsuhashi S., Masuda H. Content Production Technology on Hybrid Log-Gamma. Proceedings of the SMPTE 2017 Annual Technical Conference and Exhibition.

[4] Rissanen J., Langdon G.G. (1979). Arithmetic coding. IBM J. Res. Dev..

[5] Chen Y., Murherjee D., Han J., Grange A., Xu Y., Liu Z., Parker S., Chen C., Su H., Joshi U. An overview of core coding tools in the AV1 video codec. Proceedings of the 2018 Picture Coding Symposium (PCS).

[6] Sullivan G.J., Ohm J.R., Han W.J., Wiegand T. (2012). Overview of the high efficiency video coding (HEVC) standard. IEEE Trans. Circuits Syst. Video Technol..

[7] Chen X., Zhang Z., Ren J.S., Tian L., Qiao Y., Dong C. A New Journey From SDRTV to HDRTV. Proceedings of the IEEE/CVF International Conference on Computer Vision (ICCV).

[8] Kim S.Y., Oh J., Kim M. Deep SR-ITM: Joint Learning of Super-Resolution and Inverse Tone-Mapping for 4K UHD HDR Applications. Proceedings of the International Conference on Computer Vision.

[9] Kim S.Y., Oh J., Kim M. Jsi-gan: Gan-based joint super-resolution and inverse tone-mapping with pixel-wise task-specific filters for uhd hdr video. Proceedings of the AAAI Conference on Artificial Intelligence.

[10] He G., Long S., Xu L., Wu C., Yu W., Zhou J. (2023). Global priors guided modulation network for joint super-resolution and SDRTV-to-HDRTV. Neurocomputing.

[11] He G., Xu K., Xu L., Wu C., Sun M., Wen X., Tai Y.W. SDRTV-to-HDRTV via Hierarchical Dynamic Context Feature Mapping. Proceedings of the 30th ACM International Conference on Multimedia (MM ’22).

[12] Xu N., Chen T., Crenshaw J.E., Kunkel T., Lee B. (2017). Methods and Systems for Inverse Tone Mapping. U.S. Patent.

[13] Ballestad Andrey A., Ward K.J. (2015). Method and Apparatus for Image Data Transformation. U.S. Patent.

[14] Akyüz A.O., Fleming R., Riecke B.E., Reinhard E., Bülthoff H.H. (2007). Do HDR displays support LDR content? A psychophysical evaluation. ACM Trans. Graph. (TOG).

[15] Banterle F., Ledda P., Debattista K., Chalmers A. Expanding low dynamic range videos for high dynamic range applications. Proceedings of the 24th Spring Conference on Computer Graphics.

[16] Banterle F., Debattista K., Artusi A., Pattanaik S., Myszkowski K., Ledda P., Chalmers A. (2009). High dynamic range imaging and low dynamic range expansion for generating HDR content. Computer Graphics Forum.

[17] Marnerides D., Bashford-Rogers T., Debattista K. (2021). Deep HDR hallucination for inverse tone mapping. Sensors.

[18] Liu Y.L., Lai W.S., Chen Y.S., Kao Y.L., Yang M.H., Chuang Y.Y., Huang J.B. Single-image HDR reconstruction by learning to reverse the camera pipeline. Proceedings of the IEEE/CVF Conference on Computer Vision and Pattern Recognition.

[19] Eilertsen G., Kronander J., Denes G., Mantiuk R.K., Unger J. (2017). HDR image reconstruction from a single exposure using deep CNNs. ACM Trans. Graph. (TOG).

[20] Santos M.S., Ren T.I., Kalantari N.K. (2020). Single image HDR reconstruction using a CNN with masked features and perceptual loss. ACM Trans. Graph. (TOG).

[21] Debevec P.E., Malik J. Recovering high dynamic range radiance maps from photographs. Proceedings of the 24th Annual Conference on Computer Graphics and Interactive Techniques.

[22] Lee S., An G.H., Kang S.J. Deep recursive hdri: Inverse tone mapping using generative adversarial networks. Proceedings of the European Conference on Computer Vision (ECCV).

[23] Niu Y., Wu J., Liu W., Guo W., Lau R.W. (2021). HDR-GAN: HDR image reconstruction from multi-exposed LDR images with large motions. IEEE Trans. Image Process..

[24] Yan Q., Zhang L., Liu Y., Zhu Y., Sun J., Shi Q., Zhang Y. (2020). Deep HDR imaging via a non-local network. IEEE Trans. Image Process..

[25] Dong C., Deng Y., Change Loy C., Tang X. Compression artifacts reduction by a deep convolutional network. Proceedings of the IEEE International Conference on Computer Vision.

[26] Zhang K., Zuo W., Chen Y., Meng D., Zhang L. (2017). Beyond a gaussian denoiser: Residual learning of deep cnn for image denoising. IEEE Trans. Image Process..

[27] Dai Y., Liu D., Wu F. (2017). A convolutional neural network approach for post-processing in HEVC intra coding. Proceedings of the International Conference on Multimedia Modeling.

[28] Zhang Y., Shen T., Ji X., Zhang Y., Xiong R., Dai Q. (2018). Residual highway convolutional neural networks for in-loop filtering in HEVC. IEEE Trans. Image Process..

[29] Yang R., Xu M., Liu T., Wang Z., Guan Z. (2018). Enhancing quality for HEVC compressed videos. IEEE Trans. Circuits Syst. Video Technol..

[30] He X., Hu Q., Zhang X., Zhang C., Lin W., Han X. Enhancing HEVC compressed videos with a partition-masked convolutional neural network. Proceedings of the 2018 25th IEEE International Conference on Image Processing (ICIP).

[31] Ding D., Kong L., Chen G., Liu Z., Fang Y. (2019). A Switchable Deep Learning Approach for In-loop Filtering in Video Coding. IEEE Trans. Circuits Syst. Video Technol..

[32] Xue T., Chen B., Wu J., Wei D., Freeman W.T. (2019). Video enhancement with task-oriented flow. Int. J. Comput. Vis..

[33] Yang R., Xu M., Wang Z., Li T. Multi-frame quality enhancement for compressed video. Proceedings of the IEEE Conference on Computer Vision and Pattern Recognition.

[34] Guan Z., Xing Q., Xu M., Yang R., Liu T., Wang Z. (2019). MFQE 2.0: A new approach for multi-frame quality enhancement on compressed video. IEEE Trans. Pattern Anal. Mach. Intell..

[35] Deng J., Wang L., Pu S., Zhuo C. Spatio-temporal deformable convolution for compressed video quality enhancement. Proceedings of the AAAI Conference on Artificial Intelligence.

[36] Dai J., Qi H., Xiong Y., Li Y., Zhang G., Hu H., Wei Y. Deformable convolutional networks. Proceedings of the IEEE International Conference on Computer Vision.

[37] Ronneberger O., Fischer P., Brox T. (2015). U-net: Convolutional networks for biomedical image segmentation. Proceedings of the Medical Image Computing and Computer-Assisted Intervention–MICCAI 2015: 18th International Conference.

[38] Wang X., Yu K., Dong C., Loy C.C. Recovering realistic texture in image super-resolution by deep spatial feature transform. Proceedings of the IEEE conference on Computer Vision and Pattern Recognition.

[39] He J., Liu Y., Qiao Y., Dong C. (2020). Conditional sequential modulation for efficient global image retouching. Proceedings of the European Conference on Computer Vision.

[40] Wang Z., Simoncelli E.P., Bovik A.C. Multiscale structural similarity for image quality assessment. Proceedings of the Thrity-Seventh Asilomar Conference on Signals, Systems & Computers.

[41] Kingma D.P., Ba J. (2014). Adam: A method for stochastic optimization. arXiv.

[42] Ho M.M., Zhou J., He G. (2021). RR-DnCNN v2.0: Enhanced Restoration-Reconstruction Deep Neural Network for Down-Sampling-Based Video Coding. IEEE Trans. Image Process..

[43] Xu G., Hou Q., Zhang L., Cheng M.M. FMNet: Frequency-Aware Modulation Network for SDR-to-HDR Translation. Proceedings of the 30th ACM International Conference on Multimedia (MM ’22).

[44] Chen X., Liu Y., Zhang Z., Qiao Y., Dong C. HDRUnet: Single image HDR reconstruction with denoising and dequantization. Proceedings of the IEEE/CVF Conference on Computer Vision and Pattern Recognition.

[45] Shao T., Zhai D., Jiang J., Liu X. Hybrid Conditional Deep Inverse Tone Mapping. Proceedings of the 30th ACM International Conference on Multimedia (MM ’22).

[46] Yang C., Jin M., Jia X., Xu Y., Chen Y. AdaInt: Learning Adaptive Intervals for 3D Lookup Tables on Real-time Image Enhancement. Proceedings of the IEEE/CVF Conference on Computer Vision and Pattern Recognition.

